# Foreign

**Published:** 1841-08-21

**Authors:** 


### FOREIGN.

*A Winter in the Azores, and a Summer at the Baths of the Furnas.* By JOSEPH BULLAR, M. D., and HENRY BULLAR, of Lincoln's Inn.—*London*, 1841.

*Indolence and Corpulence of the Azoreans.*—The women of all the classes above the poor keep very much at home; the men of the same ranks are neither active sportsmen nor pedestrians, nor do they indulge in any of the manly games of our countrymen, or (as far as I

have seen) in any substitute for them. Those in trade conduct it in the most leisurely way, shutting up their shops at two o'clock; and they are as scrupulous in attending to the red-letter days in the almanack as an English school-boy used to be when such events were more observed than they are now. Few ride on horseback; the majority move along upon the backs of easy-paced asses, sitting side-ways on a soft cushion. The inevitable consequence follows. Man's body is alone suited to earning his bread by the sweat of his brow, and he cannot defy that curse without a heavy punishment. If he need not earn his bread himself, he must substitute laborious pleasures: he must work harder than a post-boy, under the name of hunting; or, for mere relaxation, encounter such cold and wet, hunger, thirst, and fatigue in deer-stalking or grouse-shooting, as a half-famished North American Indian meets with who has a starving family depending on his success; or he must rise early and work harder than a labourer in toiling over ploughed ground and stubble fields, or through wet turnips and thick grass in pursuit of partridges or hares: or he must walk up and down the same street in the same country town with all the assiduity of a policeman, to the market, or his club, or newsroom: or with his wife, or for his wife: or he must play at bowls or cricket, at gardening or at navigation; if he does none of these things or similar ones, he grows fat, has indigestion, and consults doctors with the vain hope of being enabled to baffle nature with impunity for some little time longer, and after a few years of perpetual uneasy feelings, it is found that his heart is diseased, he becomes dropsical, or loses the use of one-half of his body, and is wheeled about in a chair, imbecile in mind as well as in limbs, or he becomes melancholy, and suspicious of his best friends, or by some such winding up he arrives at the last scene that ends his common-place, eventless history.

Even to an English eye, accustomed as it is to "The fair round belly with good capon lined," the prodigality of fat Azoreans is striking. It is generally thought that England has a monopoly of human fat; that the conditions favourable to its inordinate growth, such as easy circumstances, abundance of beef and mutton from rich pastures, duly moistened with strong beers or strong wines, together with a constitutional selfish quietude of disposition and capacious hereditary powers of digestion,—the combined result of many generations of generous livers,—are alone found under our constitution. The Scotch have a "lean and hungry look, they think too much;" the Irish are too excitable or too poor; the French have, it is true, enormous appetites, but how can they fatten on bread, beans, dried peas, and thin wine? You may travel through their countries and not meet with more exceptions than are enough to prove the rule. I should doubt the existence of much fat in America; the unexampled busy-mindedness there must prevent all such accumulations. But here the necessary requisites are found; the rich viands and the beer are wanting, but the climate is superior; no extremes; no cold driving the fat man to unusual exertion, nor that other extreme, intense heat, melting him into a finer form; but throughout the year, by night and by day, an equable greenhouse warmth, keeping the body, even when passive, in a genial glow, and enticing it to quietude and repose.

The Castle of Indolence might have been built here; and he, who, when smitten with the delights of leisure, declared, that if he had a son he should do nothing, and be called Nothing-to-do, should have transported him to this island. No speculations are there to vex the genial current of the soul of the indolent man, who would be richer without labouring for it; politics, instead of a daily excitement, are a monthly or quarterly one, softened by time and distance; there is no literature to set men thinking,—midnight oil is never burnt,—a face sicklied o'er with the pale cast of thought is not, and no foolish, over-careful Azoreans break their sleep with thoughts, their brains with care, their bones with industry.

They say that men who, when they arrive at the Furnas, look like huge hills of flesh, after soaking an hour a day in the very hot water, and encouraging dissolution and thaw for an hour or two afterwards, by laying upon a board covered with thick wollen cloaks, with towels wound round their heads and necks, return so slim as to be hardly recognized by their nearest friends: the baths using up their spare materials as a winter's starvation does those of a hybernating dormouse. There are now a few portly individuals, sleek-headed men, and such as sleep o' nights, who seem to be here for this reducing process. As a remedy against obesity these baths may be highly useful, for they are means likely to be employed, as they require no self-denial. Order a sensual man to take hard exercise, little sleep, and less food, and you are sure to be unattended to; but direct him to use a luxury, and he may, in following his old habits, take the advice.—*Brit. and For. Med. Rev.*

---

*Malta, considered with reference to its eligibility as a place of Residence for Invalids.* By Francis Sankey, M. D. Universitatis Melitensis.—The object of the following pages is to direct the attention of medical men, in England and elsewhere, to the advantages possessed by this island, as a place of temporary residence for such persons as require a change of climate for the restoration of their health. In speaking of Malta as a resort for the delicate in health, I, of course, must be understood to mean during the winter season only, or from the beginning of October to the end of May. I state this at once, as it seems to me to remove every objection offered by writers on the climate of Malta, who talk of the oppressive heat and depressing winds, of the four summer months.

No medical man, in the present day, would think of recommending his debilitated patient to try the relaxing influence of a southern climate during the summer.

My intention is, to state with candour and brevity, what Malta has to offer to any one who may visit it in search of health; for a delusion has very generally prevailed concerning Malta in a hygienic point of view. People are impressed with the idea that the heat here is alarmingly oppressive;—that certain winds are often harassing the constitution with their debilitating influence;—that few conveniences can be obtained for securing domestic comfort; and that it is an arid rock, where a garrison of soldiers, living in a fortress, are supplied only with their rations of beef and rum; in fact, that Byron's description of the place is a faithful picture. Byron evidently composed his lines under the influence of spleen and misanthropy; besides, the state of Malta, in the present day, is very different from what it was some thirty years ago. It is no longer a "military hot-house." The course of the last few years has brought about a rapid amelioration in the island, whereby the mode of life is almost entirely assimilated to that in England and France.

The island lies in about the 36th degree of north latitude, south of Sicily, and nearly in the centre of the Mediterranean. It rises prominently above the level of the sea, to an altitude of something more than 600 feet at its highest elevation. It presents an oblong appearance of about eighteen miles long and twelve broad. Many of its valleys and ravines are exceedingly fertile; but much of its area is barren and uncultivated.

The surface of the island is undulating. The cultivated portion is divided into terraces, and small sections, by low stone walls, concealing the richest verdure within, but presenting to the eye, when viewed from a distance, a look of utter barrenness. Woods there are none, nor any collection of trees deserving the name of a grove. The Carouba, a low, spreading, and deep-coloured evergreen tree, is scattered plentifully along the sides of the hills.

The orange and the lemon plantations are enclosed within garden walls, and form no feature in Malta scenery.

To the west of Malta is another and smaller island called Gozo; and in the intermediate strait is the small islet of Comino.

The physical characteristics of all these islands are perfectly analogous. Their population amounts to about 120,000, including the military residing in villages called Casals, scattered over the islands, and in the city of Valetta, with its populous suburbs. The city of Valetta may vie with any town on the shores of the Mediterranean, for the elegance of its construction. It is built upon a small peninsula which is situated between two arms of the sea running nearly parallel into the land, forming two magnificent harbours. The streets are broad, and intersect each other at right angles, thus dividing the houses into large quadrangular groups. The houses are solidly built of stone, of two, or at most three, stories high. The roofs being flat, and guarded by low parapets, afford agreeable promenades, whence, for the most part, you may obtain a view of the sea and of the country. The rooms are large and lofty, and are generally furnished with fire-places, a comfort introduced by the English since the island has become a British possession. The covered projecting balconies, in front of the houses, have a picturesque and striking appearance.

The streets are paved or macadamized: they are quickly dried after rain, and are kept exceedingly clean, so that nothing is suffered to remain which might produce noisome effluvia or engender disease. The inns are numerous and good. There is no longer any difficulty of obtaining commodious apartments or furnished houses; and living is much cheaper than in England.

Residences in the country, or on the sea side, with gardens attached, are to be had at short distances from the city.

The markets are well supplied with meat, poultry, vegetables, and fruit.

There is very little game; it principally consists of those migrating birds, which, in their transit to and from Europe and Africa, alight on this island. These are chiefly quail, plover, and beccafighi.

There is a plentiful supply of fish, little of which would be considered by the ichthyophagic epicure as really good.

No place can boast of a greater equality of temperature. Throughout the winter, an entire day without sunshine, or an entire day of rain, is equally rare, although it does occasionally occur.

The thermometer does not vary more than four or five degrees during the twenty-four hours. From the end of September to the end of May, the temperature is most delightful; a complete spring reigns in the island. Even in January and February, the temperature is never so low as to be disagreeable to persons coming from a more northern clime.

The following may be considered as a tolerably fair average of the thermometer during the year, taken daily at the hours of nine, twelve, and three, during each month of the year 1838.

The eight temperate or winter months.

| | Max. | Med. | Min. |
|---|---|---|---|
| October, - | 70 | 69½ | 69 |
| November, - | 65 | 64 | 63 |
| December, - | 58 | 56½ | 55 |
| January, - | 56 | 53½ | 51 |

| | Max. | Med. | Min. |
|---|---|---|---|
| February, - | 58 | 55½ | 53 |
| March, - | 59 | 57½ | 56 |
| April, - | 62 | 60½ | 59 |
| May, - | 71 | 70 | 69 |

The four summer months.

| | Max. | Med. | Min. |
|---|---|---|---|
| June, - | 75 | 74 | 73 |
| July, - | 82 | 79½ | 77 |
| August, - | 82 | 80 | 78 |
| September, - | 77 | 76½ | 76 |

In February and March, there are severe gusts of wind, which sweep over the island. In the city of Valetta, from the rectangular position of the streets, the wind gains increased force; but although violent, it is not like the keen and cutting breeze which descends from a snow-covered mountain range. The heat in summer is moderated by cooling currents of air, unobstructed by hill or forest. In this respect, Malta has decidedly the advantage over Italy and the south of France, where the changes of temperature, according to the direction of the wind, are as violent as they are sudden. The much talked of and dreaded sirocco is by no means so terrible as has been represented. On this head, much exaggeration has gone forth. Dr. Hennen, in his medical topography, and others, from Brydone to the present day, have written strange absurdities regarding this wind. The effects attributed to it have been ridiculously magnified. To it the fanciful ascribe all their morbid feelings. The imprudent, eager to palliate their indiscretions, find a ready excuse in the siroc. At some seasons of the year it is certainly unrefreshing and disagreeable, more particularly so in the months of August and September. It blows from between the east and south, and is a hot and humid wind. The atmosphere is usually overcast with a hazy vapour, communicating to everything a moist and somewhat clammy feel.

In Africa it is an exceedingly dry and rather strong wind; but when it arrives at Malta, having passed over a considerable expanse of sea, it becomes saturated with aqueous vapour. The autumnal sirocco has, doubtless, a considerable influence over the animal system; it throws a degree of inertness over both the mind and the body. This kind of langour and lassitude is felt chiefly by those persons who are of a nervous temperament, of an indolent disposition, or by those who continue, in a southern latitude, the luxurious habits of a more northern climate; but this wind rarely blows for more than one day consecutively. In the winter, it is soft and balmy. Several invalids, with dry cough, and bronchial irritation, have expressed themselves as always feeling better during its continuance. Dr. Hennen's account of Malta, in his medical topography of the Mediterranean, should be read at present with considerable limitations. His remarks are generally good, but he was certainly most extravagant when he wrote about the "implacable heat" and "showers of mud."

The range of the thermometer in the course of the year is from about 50° to 85°, and the medium temperature in the twelve months is never above 70°. For the "showers of mud," we must read dust, which, wafted before the wind, may in part be united with the aqueous vapour in the atmosphere, and thus be deposited over the land,—a thing which happens by every roadside in England.

But as we are not advocating a summer residence here to the debilitated invalid, it were vain to consider farther the errors and exaggerations of former writers. Indeed, the sources of objection either no longer exist, or their authors were misled by erroneous views.

In Dr. Hennen's time there existed a slight source of malaria in the stagnant water situated at the head of the great harbour. This place, however, has been entirely drained, so that there is no longer any spot of marshy stagnation in the island to give origin to malignant fevers.

There are no vegetable matters running into decomposition, no animal substances allowed to putrify above ground. The island is open to every wind that blows, so that there is nothing in the natural state or geographical position of Malta that necessarily generates any kind of hurtful malady.

In the able statistical reports by Major Tullock, published in 1839, from official returns, some serious errors occur. The report states that Malta is not so healthy as Britain. This report is founded on the deaths occurring in an average population of 10,000 people compared with the same number in England. It is not just to ground such statements on the deaths alone. The proper basis of the calculation would be the number of the sick; for such is the difference between the two countries, that a simple affection, which, in England, would be cured in a few days, is allowed by the natives to degenerate into serious disease. The people cannot pay for good medical attendance, and are averse to taking any sort of medicine. They have not the means of changing a scanty and unwholesome diet for more nourishing aliment. The people in the country eat the coarsest description of food, bad bread, crude vegetables, olives, and inferior kinds of salt fish. Meat they seldom touch, and wine only on festival days; and then perhaps to excess. The long fasting of forty-four days in Lent, in addition to the generally unhealthy course of diet, is another source of debility, and, therefore, a predisposing cause of disease. From these circumstances, coupled with bad clothing, and dirty habits, the people here have not sufficient energy to support disease, so that of an equal number of patients in England and Malta, a far greater proportion would recover in the former country. If due allowance be made for all the diseases which the inhabitants bring on, or continue on themselves, either by carelessness or necessity, there will not be found in the south of Europe a more healthy spot than this island. The mortality here, notwithstanding the causes above stated, cannot, in ordinary years, be calculated at so much as 3 per cent. The average number of deaths annually, according to the Statistical Report, for thirteen years, is 2577, which, on an average population of 100,270, amounts to rather more than 2½ per cent.

The existence of the predisposing causes of want of energy, impure circulating fluids, want of the means of prevention and of cure, should tend to keep the population stationary, if not cause its number to retrograde. But the excess of births over deaths is very considerable. If, then, an annual augmentation can be shown, it must be conceded that the island is a salubrious place; and this fact must prove the fallacy of all that has been advanced to the contrary.

Another most erroneous statement exists in the table of returns for pectoral complaints. This error arises from a mistake in the meaning of the term consumption, as it is applied by the Maltese practitioners. These gentlemen understand by this term, any wasting of the body, from whatever cause it may arise, such as general scrofulous glandular disease, the natural decay of age, or a frame worn out by constitutional irritation, even if that irritation be produced from accidental violence.

In the surgical department of the hospitals, all cases of death which take place from purulent discharge or constitutional irritation, subsequent to the receipt of some severe injury, are returned by the surgeons of these establishments as cases of consumption, and registered as such in the police reports. Very recently, a respectable lady, the wife of a judge, died from the exhaustion consequent to a severe burn received some time previous to her decease. This lady's death was returned as a case of consumption. A woman died of abscess of the uterus, occurring after a laborious parturition. The cause of her death was returned under the same sweeping denomination. Consumption, as it is understood here, has not necessarily any reference to pectoral disease. In the case of chronic *pulmonitis* terminating in death, where there are no tubercular deposits in the lungs, the disease may be called consumption; whereas, if these tubercles existed, the complaint would be called phthisis; so perfectly distinct are the limits of these affections in the mind of the native practitioner.

The following are the words of Dr. Bardon, surgeon of the Civil Hospital, to an inquiry as to the meaning he attached to the term consumption. "By consumption is to be understood the wasting and emaciation of the body to the utmost degree. This effect, arising from a diminished power in the digestive action, may have many primary causes; any irritation, whatsoever may be its seat, or whatever be its nature, can give origin to the state called consumption; for instance, neuralgic affections, violent and prolonged pain, continued mental emotion, and particularly nostalgia, may be followed by this wasting of the body termed consumption. The encephalon being disordered, will involve other viscera, and more especially enfeeble the digestive power. Wherever morbid action is established, consumption may follow as a consequence. It may be caused by acute, as well as by chronic disease, but more generally by the latter."

The term is so well understood here, that it is a matter of extreme surprise to me to find the person sending home these returns ignorant of the fact. Under this class, therefore, there must be assembled a vast number of diseases which are not pectoral, or if pectoral not phthisical; and this monstrous error has been adopted and continued in the official reports under the head of diseases of the lungs. There is an attempt to correct or palliate the mistake in a slight marginal note, but the calculation is made in the error, and is, therefore, necessarily false. According to the tables in that report for thirteen years, in an average population of 100,270, there died, by consumption and phthisis pulmonalis, 4149 or 319 annually. Of these cases, 2786 were not phthisical diseases, which number being deducted, leaves 1363 cases only of pulmonary consumption, or nearly 105 annually, amounting to about one in a thousand of the civil population. The whole number of diseases of the respiratory system of all kinds, for the same period of thirteen years, is returned 6664; from which, deducting diseases which ought not to be classed under this denomination, we have 3878 or 298 yearly, about three in a thousand of the whole population.

Major Tulloch, in his report, says, that a greater proportion of the British Troops die in Malta than in England; from which he concludes, that the climate is unfavourable to health. May we not rather look for a cause of this increase of mortality in the well known fact, that soldiers are generally addicted to strong drink? that the thirst produced by the heat prompts them to gratify the propensity to excess, which they can do so readily in consequence of the low price of wines and ardent spirits? To this vice we may fairly attribute the increase of mortality among the foreign troops on the Mediterranean stations. Let us compare this assertion with the report in the same work on the health of the Maltese Fencible Regiment. We observe, that the average mortality in this corps is amazingly reduced by the superiority of their food over that of the other inhabitants, as well as by the greater attention given to their health, together with their habits of sobriety. The report says, "this corps was organized in the year 1825, and is composed of Maltese, enlisted for a limited period of service, with the understanding that they are not to be employed off the island."

By an extract from the War Office returns, it is shown, that for eleven years the strength of this corps averaged 515 men, of whom 4 7-11ths have died annually, being at the rate of nine per thousand, which is less, by one half, than the mortality among the foreign troops, all men of the same age, vigour of constitution, and placed under precisely similar circumstances. How shall we attempt to account for this marked difference? I think we may justly affirm, that it arises from the different habits of the men. It cannot be, as the author of the report would infer, that, as the Fencible soldier is indigenous to the climate, he enjoys greater immunity from disease: for if so, we should find, that the foreign families resident on the island, and those in the civil employ, would suffer in proportion to the troops of the line, which is not the case.

The author of the report goes on to say, that there are two circumstances, independent of climate, to which exemption from disease, which these native soldiers enjoy, is mainly attributable. The comparative abstinence from those excesses to which the British soldier is addicted, and the fact, that many of them being married men, are therefore likely to be free from those diseases which constitute so large a number of the admissions into our hospitals. Thus the reporter refutes, in a manner, the errors of his own statement, and proves the truth of the assertion, that the mortality among the foreigners is more dependent on their habits than on climate, and that the number of deaths among the general civil population, is owing more to their necessities than to any atmospheric or telluric influence.

I do not, of course, pretend to affirm, that the climate and air of Malta possess any curative virtue for those persons, whose organic diseases have placed them beyond the power of recovery elsewhere. Where tubercular disease has proceeded so far as to make extensive ravages in the pulmonary tissue, it is ever useless and cruel to send such unhappy persons far from their homes and sympathizing friends, to die among strangers in a foreign land.

At the present day it is pretty generally understood by medical practitioners, that such removal is mischievous, both from the fatigue attending the journey, and chiefly that the change to a warmer latitude, by increasing the irritability of the hectic sufferer, and augmenting the nocturnal perspirations, would farther reduce the strength of the patient, and hasten the catastrophe. Yet sometimes the medical attendant trusts that disease has not committed the ravages he suspects, or he is induced to yield to the wishes and solicitations of the patient, whose hope of recovery often appears commensurate with the fatal tendency of his complaint.

But to persons who are suffering from constitutional debility, who, either from conformation or accidental circumstances, are strongly predisposed to pulmonary disease, or are in any state of cachexia, unattended with considerable organic lesion, a residence in a southern latitude, for three or four months during the winter, is highly useful; and I think Malta offers advantages to such persons, equal to those of any other place. Among those advantages we may enumerate,—the voyage by sea, when land travelling would be too fatiguing; the facility of conveyance from any part of the surrounding continents; the novelty of the scene, so different from any thing which Europe can offer; the living under the British flag, in a British possession; the many comforts that may be commanded; the power of obtaining the assistance of English medical practitioners resident in the island, as well as those of the staff of the army, or navy; and a climate where, on every day of the year, for some hours of that day, exercise in the open air, or gentle boat exercise, may be taken, so essential to the re-establishment of health.

Another advantage which Malta possesses over almost every other place, is the great facility which the invalid has of getting away, should he find, or fancy he finds, the climate uncongenial to his constitution.

The almost daily arrival and departure of English, French, and Italian steamers, will enable him to try other countries, to make excursive trips, and again to return,—thus frequently changing the air and scene, both of acknowledged use to the infirm.

I have known persons, whose health has been considerably improved by residing for a while in Malta, and making short voyages to the neighbouring states.

The great benefit derived by numerous invalids, that for several winters past have visited Malta, ought to silence the objections of those whose opinions are founded on the erroneous data in the otherwise excellent report of Major Tulloch, or in the medical topography of Dr. Hennen.

The residence of her Majesty the Queen Dowager Adelaide, during the winters of 1838–9, has tended greatly to give a deserved celebrity to this island: and her liberality has added a handsome church to its public buildings.

To the mere seeker after pleasure, it may be said to be wanting in matters of deep interest or excitement. There is little extent of country, and diversified scenery; no extensive lawns, nor woods, nor streams of water; and no great variety of rides or walks. Your exit from the city must be by the same fortified gate, over the same drawbridge. There is little room for the display of brilliant equipages; and society is not so artificial as in the great European capitals.

In the way of amusements, Malta can offer little beyond the following:—namely,

A theatre for Italian music, with very respectable vocal performers, and an excellent orchestra; public promenades, where the military regimental bands play at certain hours of the day; a garrison library, and a public government library, which are accessible to every one; a subscription club, with mercantile subscription reading-rooms; numerous public balls during the winter; saddle horses, or close and open carriages, to be had at all times on the most reasonable terms; and yachts and numerous smaller pleasure boats for excursions round the island.

Valetta cannot be said to be a monotonous, or a dull place. The presence of the garrison and the fleet; the influx of strangers from all nations; the continual arrival and departure of ships of war and packets, keep the mind amused by the continually shifting scene, and render Malta equal to any place in liveliness of aspect.

In putting down the preceding remarks, intended to direct the attention of my professional brethren to Malta, as a desirable place of residence for the invalid during the winter, equal if not in most cases preferable, to any other place in the south of Europe, I may have been prompted by the laudable desire to favour this British possession; but I trust it has been done with a strict regard to justice, without either indulging in extravagant praise of this, or disparaging the merits of other places; a course of conduct which, I feel convinced, would injure my cause with every discerning person.—*Edinburgh Med. and Surg. Jour.*

---

*On Dilatation by Fluid Pressure in Stricture of the Urethra.* By James Arnott, M. D.—Although our knowledge of the pathology of stricture of the urethra has been much extended by the labours of Hunter and others, the treatment of this very common and distressing disease differs at the present day in no very material circumstance from that which was followed two hundred years ago. In the works of Wiseman, published in the reign of Charles II., the various practices now had recourse to will be found described. He mentions the use both of metallic and soft bougies; the application of caustic is noticed, a practice which was revived by Hunter; and cases are related in which the operation of opening the urethra behind the stricture was performed, instead of puncturing the bladder—an expedient of which the late eminent Sir Astley Cooper has been deemed the original proposer.

Unfortunately, this stationary condition, during the progress of almost every other department of surgery, has not proceeded from the treatment of stricture having attained perfection. On the contrary, it is acknowledged to be an opprobrium of the art. The means employed are admitted, by conscientious and intelligent surgeons, to be, in almost every case, but palliative; and although stricture may generally be much relieved by such means applied from time to time, it cannot be denied that the irritation which accompanies organic changes in a part of so much sensibility as the urethra, will, by long continuance, often produce other disease in the neighbouring organs of the urinary and generative systems, which is sure to embitter, if it does not shorten, the life of the sufferer.

It is now many years since I introduced to the profession an account of practices in the treatment of stricture which I had had sufficient experience to recommend as substitutes for the very imperfect and sometimes hazardous measures in common use. But because the apparatus recommended was of rather a complicated description, as compared with that usually employed, and because part of it was constructed on mechanical principles, with which surgeons generally were not familiar, it has either not been used at all in this country, (where the French modifications of plans of treatment I had proposed in impervious stricture, and of a new method of applying caustic, are almost unknown,) or in so imperfect and erroneous a manner, as to disappoint expectation.

The purpose of this paper is to describe a modification, which I have lately contrived, of the instrument employed in the dilatation of stricture, combining the essential requisites, for general use, of simplicity of construction and easiness of application; and I cannot doubt, from its great and manifest superiority over the means commonly had recourse to, in the degree of relief afforded by it, and the safety and quickness with which this is obtained, that it only requires to be known to be immediately adopted.

Dilatation of stricture has been effected in two ways: by instruments which operate on the principle of the wedge, opening the constricted part as they advance in the canal, of which description are bougies and sounds; and by instruments which are themselves capable of distension, and which, by being made to enlarge in diameter whilst within the stricture, exert their dilating force from the centre directly outwards, or, as it may be termed, eccentrically. Amongst the principal advantages of eccentric dilatation over that of the wedge, when effected by a proper apparatus, are—that instruments so operating, having no tendency to stretch or tear the urethra in front of the stricture, by pushing on the stricture after having passed partially through it, the surgeon is enabled by their means to use greater force (if required) with safety, than with rigid bougies and sounds, which have this tendency; that there is no danger, from the opposition to the passage of the instrument being erroneously attributed to the stricture instead of the wrong direction of its point, of the surgeon's piercing the side of the canal, and causing effusion of urine, or false passage; that the dilatation being effected without irritation from friction, and following the yielding structure, may be rapidly made; that the whole of a long stricture is dilated at once, or several strictures are acted upon at the same time, instead of the action being nearly confined, as in the case of the bougie, to the front or face of the first stricture; and that, from the power of enlarging the instrument in the interior of the canal to any size, the dilatation of the diseased part may be carried to any greater extent than the diameter of the outer orifice of the urethra, so as to afford the best means of effecting a permanent cure.

The apparatus used for dilatation, on the principle just explained, consists essentially of a strong membranous tube of fixed dimensions, which is placed, in its empty or collapsed state, within the stricture, and then injected with fluid. I have used such a fluid dilator, with various modifications, according to peculiarities of cases. The form of instrument most easily constructed and applied, and which has not as yet been described, is merely a varnished silk tube of the required diameter, and of a length to extend from the orifice to a little beyond the stricture, closed at one end, and having a small metallic connecting piece at the other, into which the injecting syringe may be screwed. This tube, by means of a slight coating of waxy composition, is, for the purpose of passing easily, rolled into the form of a common plaster bougie; and when it is not required to be of very small diameter in its collapsed state, the requisite stiffness may be given to it by rolling it upon a small catgut or stilet. A woven silk tube properly varnished would be perfectly water-tight; but this is of less importance, as a thick mucilaginous liquid will not escape but very slowly from a very imperfect tube made by sewing together the edges of a riband. This instrument, which may be described as a dilatable bougie, is as durable, and may be made at as little expense, as any instrument used in the treatment of stricture.

In keeping up distension, by means of a dilator rendered impervious to fluid by gut or caoutchouc, instead of the stopcock recommended in former instructions on the subject, a contrivance may be employed for fixing the piston of the syringe, when the required degree of pressure has been made, as by a cord passing through the ring at the end of the piston rod, or by a screw. When the piston is depressed by a screw (which may constitute the piston rod) the patient can himself increase or moderate the pressure with the greatest facility. If the distensible tube be made of strong silk, it may be thus gradually distended until it becomes as hard as a cylinder of wood. A connecting flexible tube of silk and caoutchouc between the metallic part of the dilator and syringe, prevents any jerking motion of the instrument in the act of screwing on the syringe, and is a convenient index of the degree of pressure applied.

In other applications of the fluid dilator, as in the cure of stricture of the rectum, and in the operation of slowly dilating the male or female urethra for the extraction of calculi, a long connecting tube of this description, bringing the screw which regulates the pressure conveniently to the hand of the patient, would render the apparatus very complete. I have shown in the appendix to the late edition of my work on Stricture and Stone, that the advantage of *slow* dilatation of the male urethra must have frequently occurred to the operators by the Marian method, who professed to follow nature in their proceedings, as it has occurred independently to several surgeons of late years; but that the want of any instrument which could fulfil the indication must have prevented the success of any attempt of the kind. The equable, elastic, and controllable nature of fluid pressure, makes a dilator, judiciously constructed on this principle, incomparably superior to any other means that has been employed for the purpose; and furnishes us with a method of extracting urinary calculi, which, if I am not much deceived, will soon supercede the present painful and dangerous operations.

When stricture is to be dilated beyond the diameter of the orifice of the urethra, it is necessary to modify the instrument which has been mentioned above. The distension may be confined to the diseased part by placing a wide silk tube within another shorter tube of smaller diameter, or by passing it through a wide silver or elastic tube previously inserted as far as the stricture. In cases of very narrow stricture, only admitting instruments of the smallest size, a dilator may be passed through such a conducting tube, consisting either of a single or double piece of narrow gut dried in a compressed form, or of a silk tube rolled upon itself, and rendered sufficiently rigid by means of thick mucilage. It is unnecessary in these cases to have a distensible tube of the whole length of the conductor; a small bit tied upon the end of a long flexible tin tube, connecting it with the syringe, is sufficient.

In mentioning this mode of conveying a small instrument to narrow strictures by means of a condncting tube, I am led to notice a controversy which has been continued through several late numbers of the Medical Gazette, respecting the invention of what has been termed "the compound catheter." Dr. Buchanan, who claims the originality of this proposal, does not appear to be conversant with the modern French writers on urinary diseases, or he would have found that the plan of conducting small instruments through others of larger size is noticed in most of the works on that subject which have appeared in France during the last twenty years. But it is contended that Dr. Buchannan's instrument is more than a mere modification of former suggestions; that the principle of it extends further: the smaller instrument, it is said, is not only conducted, but a way is prepared for it through the stricture by a dilatation effected by the pressure of the ends of the outer canulæ. Had a reference been made to the work from which M. Ducamp borrowed so liberally, instead of the treatise of M. Gerdy, who in this matter professes merely to follow Ducamp, it would have been discovered that there is no greater novelty in this idea of previous dilatation, than in that of conducting. In my Treatise on Stricture of the Urethra, (p. 133, 2d edition,) the following is mentioned amongst other means to be resorted to in cases of difficulty;—"The plan which I have recommended above, of passing a large canula (which in this case has a rounded end) enclosing an instrument down to the stricture, is very applicable here; pushing the canula against the stricture opens it, while the small bougie or catheter within is ready to be passed through."

*Lon. Med. Gaz.*

---

*New Method of treating Cases of purely functional Neuralgy. Exhibition of the Galvanic Factors.* By Robert Dick, M. D.—Although in my work, entitled "Derangements, Primary and Reflex, of the Organs of Digestion," I have by no means overlooked those forms of ventro-intestinal disease, which either apparently consist purely in, or are accompanied and principally characterised by, lesions of innervation, yet I propose, in the following paper, to direct attention more particularly to them, chiefly with the view of submitting to the profession a mode of treating such cases which, whatever opinion may be formed of its theoretical merits, will (as I can affirm from repeated observation) be found practically useful in many instances.

The palpable structural degenerations and deformations which present themselves to the advanced stages of many diseases, far of course from constituting primary lesions, are but the last of a long series of morbid changes, and indicate, usually, the helpless prostration of nature's conservative and reparative powers. The limitedness of our senses, our still most imperfect knowledge of the laws and habitudes of living parts and organs, permit us to perceive only the nearest and grossest links in the chain of morbid relation and succession. The *commencement* of that chain almost always (may I not say in every case?) eludes our nicest scrutiny, and baffles our attempt even to conjecture its manner and its whereabout.

Much more of our treatment of all diseases than perhaps any of us would care to admit, consists in the observed results of mere *tentative* experiments (as contradistinguished to the products of regular *induction*) by which certain useful information and certain effects have been developed, often unexpectedly, on our part. But this is no stigma upon us, or upon our art, since it is owing to the limited nature of our faculties of knowledge of observation, as assigned by the Creator, and to the complex and abstruse nature of the subject, to wit, the human body, which the art of medicine regards.

The lesions of the nervous system, in its sensibility, seems more naturally and justly the province of empiric or tentative treatment than any others. Indeed, in many of the lesions now named, the treatment can be, in the first place, nothing else than empiric.

It is probable that lesions of sensibility, special or common, either constitute the earliest form of all disease, or else accompany it. This view, I found on the fact of that law, that nature has appointed the sensation of pain or uneasiness, as a warning indication that our organs are not performing their functions aright; that something is to be avoided or rectified on our part. It is obvious, then, that to further this useful end, lesions of sensibility must be, at least, contemporaneous with the most incipient morbid movements. We may even suppose that such lesions of sensibility ante-date such movements; since the same prospective regard, on nature's part, by which pain is made to announce to us the existence and continuance of injuries *actually* incurred may be supposed to be appointed, and to be fitted to intimate to us their approach, and *merely threatened* incidence.

If it be so, then a practical inference is deducible, namely, that whatever removes morbid sensibility is, so far as rational induction and the present state of our knowledge allow us to conjecture, the means most fitted to avert those more palpable lesions, of which morbid sensibility is the warning percursor, the symptom, or the consequence.

Some of the most perplexing and intractable complaints which physicians are called to treat, especially with patients (of both sexes) in certain stations of society, and accustomed to certain modes of life, are plainly referrible to pure but subtle affections of the nervous system. All the vital organs, as the lungs, heart, digestive viscera, and even the brain and spinal cord, also, as regards their more ordinary functions, may be acting, so far as we can discern, with perfect or tolerable efficiency. Yet anomalous pains of indescribably various character, evidently, however, not dependent on inflammation; local spasm, tremors, ventral pulsations, singular affections and illusions of the cerebro-sensal organs, feelings of heat and chilness; now, great sensibility of touch, and now the want of it, states best expressed by the French word *malaise;* with morbid conditions, both of the intellectual operations and moral affections, are vividly complained of, and often notably embitter life.

In other cases, there are nerve-aches (for no more than *aches* they obviously are) in the stomach and in the intestines—often, at the same time, in the thoracic and abdominal walls, about the site of the heart, and in the tract of the chief nerves generally, but more particularly, according to my observations, in the course of the vagi, and phrenic nerves about the root of the neck.

These, and similar symptoms, which often call for treatment, are, as I have already remarked, not due, in a great many cases, to inflammation, (understanding by that word what it is commonly meant to signify,) either of the muscular tissue, or of the nerves themselves, or their investments; to no change (appreciable at least) in the structure of those parts, but to some augmented or diminished power, or some perversion in the function of sensibility, common or special.

In such cases, it is often noticeable, that any measures addressed to the digestive organs, and designed to act peculiarly on these, either do not relieve the seemingly anomalous symptoms above detailed, or else exasperate them. I say, measures designed to act specially on the digestive organs, by which I mean evacuants of all sorts, purgative or emetic. For, from such medicaments are to be distinguished those which, although taken into the stomach, are intended to operate on parts and organs remote from it. Nor are even the mildest alteratives ordinarily of any use in such cases. As for emissions of blood, by leech, lancet, or cupping-glass, or for blistering derivants of any sort, these are either useless or injurious.

The cases now referred to are, perhaps, the most perplexing that occur in practice, and every medical man has, I believe, at one time or another, earnestly taxed his ingenuity to find some method of rationally and effectually treating them.

I have always thought that such cases did best under *tonic* treatment. I admit, readily, the vagueness of this term, and the extreme difficulty of defining what action or influence, exerted by medical substances it is, that constitutes what we call their tonic effect. It were not suitable to enter here, abstrusely or at large, on a discussion of this point. The general understanding of the profession upon it is sufficiently precise for my present purpose.

It occurred to me that if it were possible to introduce into, and, as it were, to develope through the body, galvanic action, and that in a more permanent and pervading way, than can be effected by shocks of the galvanic battery, some striking and useful results might be anticipated.

I shall presently proceed to detail, in the briefest manner, my mode of procedure, and the results: here only remarking, that the cases being those of persons suffering from morbid states, called in ordinary phrase *nervous*; that is without any *overt* lesions to which those states could be referred, were necessarily characterised by sameness, except in so far as regarded the ages, sex, temperament, previous history, and treatment of the patients.

It occurred to me, then, to try what would be the effect of the *simultaneous administration* of the ordinary galvanic factors, zinc, copper, and nitric acid. I carried out this project, in various experiments, as follows:—

A lady, unmarried, about 40 years of age, of florid temperament, delicate, and long subject to the more mitigated but functional forms of dyspepsia, complained much of a gastro-dynia, which she described as being between an aching feeling and a feeling of the stomach being gnawed or eroded. All her functions were correct. This sensation was most perceived when the stomach was empty. About noon, one day, I caused her to swallow some minute filings of zinc and copper, and about half an hour after I gave her twenty drops of dilute nitric acid. On taking the acid, relief almost immediately followed on this occasion, as subsequently on others, when she had recourse to the same means.

Another lady, under the same treatment, for a somewhat resembling gastralgia, was averse to the use of the acid, from a fear that her teeth would suffer. This person assured me that the acid was not necessary, since, having delayed taking it on one or two mornings, she found (whilst standing) relief from her stomach ache. I noticed the same thing in other cases. Can it be that the hydrochloric, or some other acid of the stomach, supplies, in such cases, the place of the nitric?

I found the same means successful in several other little cases, which I, therefore, do not think it necessary to detail. I remarked, however, that these means, if employed either immediately before or after a meal, partially or totally failed.

Such, then, was the manner in which I used this remedy against the *local* affections, the various gastro-dyniæ. In nervous cases of more *systemic* character, the mode of treatment was somewhat different.

In these cases one of the salts was given by the mouth, the other administered by inunction. Sometimes the one salt was employed in the one form, and, anon, the other, on the contrary.

If the inunction was kept up *until a metallic taste was distinctly perceived by the patient*, and the other salt was then administered, for a day or two, internally, the tonic effect of the remedies, or, at least, their power of controlling the peculiar lesions against which they were directed, was unusually very apparent.

A gentleman, unmarried, about 48; a lady, unmarried, about 42; a gentleman, unmarried, about 36; a gentleman, married, about the same age; a lady, four years married, but without family, about 30 years of age; a lady, unmarried, about 22; a housemaid, about the same age; all of whom were subject to what, with perhaps unavoidable vagueness, are called *nervous* complaints, were notably relieved by the above means.

The oxide was the form in which I employed the zinc internally, in doses of three or four grains, twice or thrice daily. The unguentum zinci was the form in which I directed externally.

The sulphate was the form in which I ordered copper by the mouth, in doses from half a grain to three grains; gradually augmenting the dose from the smaller of these quantities to the greater. In one or two cases, subsequently, I tried the ammonio-sulphate of copper, and with success.

The external form was the unguentum subacetatis cupri of the Edinburgh and Dublin Colleges. Of course, in order to admit of its being persistently used in *inunction*, it required very considerable dilution with lard.

Since I engaged myself in the practical experiments and observations, the results of which form the subject of this paper, I notice that Kaemtz, in Schweigger's Journal, has shown that dry, but efficient galvanic piles, may be constructed from organic substances, without any concurrence of metals. It is ascertained by him that—

Soda is positive, in reference to mutton fat.
Yeast " " common salt.

Will this fact explain the occasionally striking restorative effects of yeast? namely, in consequence of its contact in the stomach with the hydrochloric acid, or in the vessels, with some of the salts of the blood.

Bullock's blood is positive in reference to belladonna, &c. &c. &c.

The electrical relations of several other substances have been determined by this gentleman. These experiments may, if prosecuted on the principle which it has been the object of this paper to promulgate, furnish new hints in the administration and combination of medicines, and develope very novel results. Articles of diet and medicaments may be mutually assorted, so as to produce useful effects. Who knows but that the particular efficacy of particular forms of food may not be owing to their exerting some influence of this kind? It is not to be doubted that the greater tonic powers, and more energetic action of the metallic salts, is due to their peculiar aptitude to be the agents or vehicles of chemical and galvanic actions.

I make no attempt to explain further, than has been incidentally done in the course of the paper, the modus operandi of the means now detailed. It is enough for me to have satisfied myself, as I have done, that the plan is *practically* useful; and that, in *many* cases, more benefit will be found unquestionably to result from the simultaneous or related use of the galvanic factors above named, than is found to follow their separate and unconnected exhibition.

I would further observe, that although several cases did well without nitric acid, yet that in general its administration should not be omitted.

I have, at present, several very suitable and interesting cases under treatment. If, in the course of it, I should notice any, particularly worthy of attention, I shall communicate them through the pages of this valuable Periodical.—*London Lancet.*

---

*On the Subcutaneous Division of the Pronator and other Muscles of the Hands and Fingers.* By Dr. P. Doubovitski.—The only point of practical importance in this case, which is related at a most unnecessary length by the patient, who, though a doctor, suffered himself to be grossly maltreated for a fracture through the condyles of the humerus, is, that it is not safe to divide tendons that run in synovial sheaths. Two out of four of the tendons of his fingers thus divided failed of reunion, and he lost the power of moving the corresponding phalanges. He knows of other cases in which the same untoward result followed similar operations; and he justly observes that when under such circumstances tendons about the feet have seemed to reunite, it may have been that other muscles have performed the actions that properly belonged to them, and that the success of the operation has consisted, not in the restoration of the action of the contracted muscles, but only in the giving to the distorted foot its proper form.—*Brit. and For. Med. Rev.*, *from Annales de la Chirurgie.*

---

*Remarkable Case of Bronchotomy from a Bean in the Air-passages.* By M. Pescheux, of Verneuil.—The principal point of interest in this case is that during this operation a small artery in the neighbourhood of the circo-thyroid membrane was divided, and the child was immediately threatened with suffocation, from the effusion of blood into the trachea. The child voided blood by the mouth and nares, ceased to respire, and became cold, when M. Pescheux instantly applied his mouth to the wound, and sucked out the blood from the trachea for three or four minutes, and being fatigued M. Aury took his place. Some instants afterwards they cauterized the wound and with a female catheter passed into the trachea insufflated the lungs. In a few minutes the child revived, its respiration gradually became regular, and after some cold water had been thrown over it, it looked at the surgeons as though nothing extraordinary had happened. Although the bean could not be found, and was not expelled till five days afterwards, the child perfectly recovered.—*Ib.*, *from Gaz. Méd. de Paris.*

